# Unlocking the Potential of Electronic Health Records for Health Research

**DOI:** 10.23889/ijpds.v5i1.1123

**Published:** 2020-01-30

**Authors:** S Lee, Y Xu, AG D&apos;Souza, EA Martin, C Doktorchik, Z Zhang, H Quan

**Affiliations:** 1 Department of Community Health Sciences, University of Calgary; 2 Centre for Health Informatics, University of Calgary; 3 Analytics, Alberta Health Services

## Abstract

Electronic health records (EHRs), originally designed to facilitate health care delivery, are becoming a valuable data source for health research. EHR systems have two components, both of which have various components, and points of data entry, management, and analysis. The “front end” refers to where the data are entered, primarily by healthcare workers (e.g. physicians and nurses). The second component of EHR systems is the electronic data warehouse, or “back-end,” where the data are stored in a relational database. EHR data elements can be of many types, which can be categorized as structured, unstructured free-text, and imaging data. The Sunrise Clinical Manager (SCM) EHR is one example of an inpatient EHR system, which covers the city of Calgary (Alberta, Canada). This system, under the management of Alberta Health Services, is now being explored for research use. The purpose of the present paper is to describe the SCM EHR for research purposes, showing how this generalizes to EHRs in general. We further discuss advantages, challenges (e.g. potential bias and data quality issues), analytical capacities, and requirements associated with using EHRs in a health research context.

## Introduction

Electronic Health Records (EHRs) are systemized collections of patient health information and documentation, collected in real-time, and stored in a digital format [1]. EHRs were originally designed to facilitate clinical decision-making regarding health care delivery for individual patients, and to improve the quality of care. EHRs have seen rapid deployment in health care worldwide over the past decade. Both Canada and the U.S. saw increases in EHR adoption, but the rate differed by provinces in Canada [2,3] and between health systems within states in the U.S. [4,5]. EHRs have historically been used mainly within acute-care settings, but primary-care settings are increasingly adopting them as well. Despite the increase in EHR adoption for healthcare delivery, researchers have used these systems in a limited capacity. Presently in Canada, research facilities using EHRs are localized to primary care and specific institutional sites.

In Calgary, a city-wide inpatient EHR system called AllScripts Sunrise Clinical Manager™ (SCM) has been in operation since 2006. SCM covers four acute-care facilities (Foothills Medical Centre, Rockyview General Hospital, Peter Lougheed Centre, and South Health Campus) and one pediatric facility (Alberta Children’s Hospital). These five facilities provide health care coverage to 1.4 million people living in the Calgary Health Region, and will additionally capture those accessing care in Calgary from surrounding rural regions. Since its inception in 2006, SCM has collected longitudinal inpatient health data on 5,469,761 million individuals. This number represents any contact with Calgary hospitals (including emergency department (ED) visits), so do potentially include out of Calgary and out of province visits as well as hospitalizations. Therefore, this SCM EHR system is a comprehensive source of population-level inpatient information.

On April 1, 2009, all regional health authorities and boards across Alberta were amalgamated into Alberta Health Services (AHS). The SCM governance was transferred to this single provincial health authority. Therefore, SCM is managed by relevant AHS departments (e.g. business intelligence, privacy office, information systems) for clinical operations and IT system management. AHS has developed and instituted protocols (e.g. research ethics, data disclosure agreement and research administration agreement) to allow health research activities using AHS data, but this process had not included SCM EHR until recently.

Recently, AHS announced implementation of ConnectCare (using EPIC software), which offers a province wide EHR system. There is a growing need to understand EHR, ultimately allowing researchers to leverage the data to optimize patient care through precision medicine and precision public health. Toward that end, AHS has partnered with Centre for Health Informatics (CHI) at the University of Calgary, to work together to apply our knowledge to ConnectCare when it comes into operation.

To date, population level EHR research is lacking, and there is a need to advance on this frontier. We are using SCM as an initial base to explore and understand EHR systems for health research. Further, we will discuss the system architecture (back-end and front-end data and its users). The current review will explore analytical and administrative challenges with using EHR data for research, and includes an example application of risk adjustment analysis in the context of precision medicine and precision public health. This work provides a roadmap for research using clinical information systems and discusses concepts that are generalizable to most EHR systems.

## EHR Back End: System Architecture

There is an intricate relationship between the front-end users and the back-end of EHR systems. Information is entered at the front line by health care providers and workers, including physicians and nurses. The front-end users are asked to enter their data in various ways, such as entering structured field information (e.g. drop-down menus, numerical fields, checkboxes, radio buttons) or writing free-text documentation. This can include discharge summaries and multidisciplinary progress notes that document patient history, clinical examination, and patient progress throughout the hospitalization. The EHR client/server structure records timestamps for all patient transactions, enabling the system to track outcomes and patient care processes (e.g. recording physician orders, vitals, patient consent or refusal). Hospital protocols that are relevant to patient care, such as patient isolation protocols, are implemented in the EHR system using triggers and warnings.

To ensure interoperability between EHR systems built by different vendors, international technical standards (e.g. International Standards Organization 18308: Health informatics - Requirements for an Electronic Health Record Architecture) ensure that basic technical documentation is broadly consistent across systems [6].

**Figure 1: Flow Diagram Depicting the Data Flow from the Front End to the Back End of SCM EHR system.**
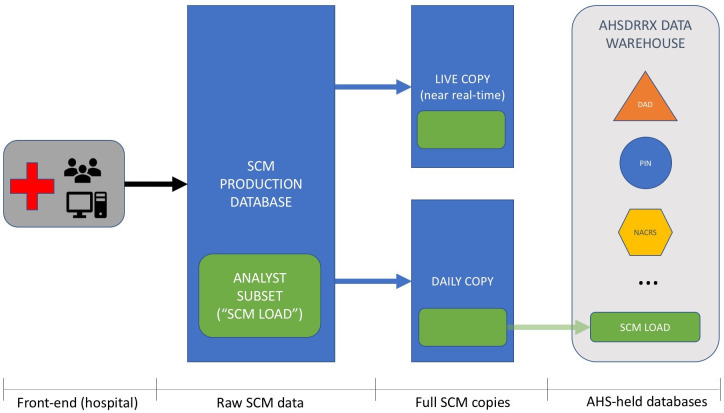

SCM is configured as a standard client/server application. Data entered from the front end is fed directly into a Microsoft SQL Server database within Alberta Health Services’ data warehouse. In addition to the main production database, several additional SCM database copies are used for various purposes (Figure 1):

1. **Live Copy:** an almost real-time replication of the SCM is available, that holds data just a few seconds or minutes behind the production database. This replication database is used for in-system reports for active patient care. Access can be granted to parts of the replication database (or even the production database) for reporting outside the system. For example, it is necessary to report on real-time data for the Emergency Department Wait Times app/web site, and access to the Live Copy SCM would be essential.
2. **Daily Copy:** a copy of the SCM production database is made once a day, between 4am and 6am. This database is primarily used for non-critical reporting and troubleshooting within the IT department at AHS. Some analysts outside of IT also use it for analytic reporting. This is a complete and exact copy of the SCM production database, and contains all free-text records. Access is generally restricted.
3. **Analyst Copy:** AHS Analytics loads a select amount of data to the Oracle Alberta Health Services Data Repository for Reporting (AHSDRRX) data warehouse. Alternatively described as ‘SCM LOAD,’ it is the warehouse that includes many other data sets including administrative data (e.g. Discharge Abstract Database, National Ambulatory Care Reporting System, Pharmaceutical Information System, etc.). This version is most familiar to analysts outside of IT. It contains only a subset of SCM tables, and is copied from the daily copy of SCM (i.e., # 2 above).

In addition to the above, SCM data flows into various other schemas within the data warehouse where it may be more analyst-friendly, or go through other validation. For example, there is an ED Visits table, which includes ED visit data from both SCM and other provincial systems. This table is in a format that is much easier to work with than the raw transactional tables. 

## Front-End EHR: Health care Workers

In Canada, clinicians input structured or unstructured information based on the patient visit into EHR for care documentation purposes. EHRs are then coded into a universal health language called the International Classification of Diseases, 10th revision, with Canadian enhancements (ICD-10-CA). 

### Structured EHR Components 

Structured data refers to types of data where the format was predetermined through an existing schema. These data are captured via structured data entry systems (SDES) on the front end [7]. Often, structured data are embedded within unstructured fields. Healthcare providers and workers often convert unstructured patient information into a structured format for easier information flow.

Typical EHR systems, including SCM, contain many structured data fields (Table 1) that use controlled fields such as problem lists, diagnoses, procedures, vital signs, medications, lab results, billing codes, demographic and other administrative data. These data are typically recorded in a long-form table within a relational database.

**Example Elements of Entered Structured Components:** 

Category	Examples of elements

Patient demographic data	Birth/death dates, first/last names, religion, gender, marital status, most recent primary provider name
Information about free-text documents	Created/authored/modified datetimes, document type (e.g. flowsheet, structured note)
Information about allergies	Allergen name/code, type (drug, contact, etc.), status (active/inactive), level of confidence (confirmed, suspected, etc.)
Information about health issues	Similar to allergies
Information about locations	Type (bed, room, etc.), facility
Information about orders	Created/modified dates, name, requester, person who entered, request date/time, frequency, status (active, completed, cancelled, etc.)
Information about medication orders and prescriptions	Route (IV, PO, etc.), dose (upper/lower limits), drug name, drug category, prescription amount, dose, frequency, duration, number of refills, modification history, deactivation/discontinuation dates
Information about providers	Role (family, attending, referring, etc.), start/end dates, status (active/inactive)
Lab/test results	Name, result, result status, order ID, historical results, reference values (upper and lower limits), whether abnormal, first/second/third level categories
Information about visits	Admit/discharge date/time, chart numbers, status (admitted, discharged, etc.), type (ED, I/P, etc.), discharge disposition, discharge location (home, facility, etc.)

There are built-in variables within the EHR to indicate clinical processes and control mechanisms, such as restricted access for specific patient records, flags for procedure receipts, and isolation status. Consider inpatient medication as an example. In the context of inpatient medication, front-line clinical and healthcare workers typically see timestamps corresponding to when a medication was ordered by a physician. Timestamps will also be made for when that particular order was fulfilled by the pharmacy, and administered to patients at bedside.

To date, structured data within EHR systems have been used in a limited capacity in research to power a wide array of data tools for end-users [8, 9,10]. For example, these data have been used to populate case reports for disease surveillance [11, 12]. Health system administrators can use structured information from procedure and diagnosis codes, as well as structured outcomes data, to evaluate and improve patient safety [13, 14]. The volume and variety of data within EHR have led to the use of machine learning techniques [15, 16]. To our knowledge, most statistical methods and machine learning algorithms either require structured input, or include some mechanism for converting unstructured data into structured input as part of the analytical pipeline.

While from a research perspective it would be ideal for most or all EHR data to be captured via structured fields, there are practical barriers to this, including physician resistance to SDES use [7] and lack of ability to capture contextual information [17, 18]. Hence, EHR systems such as SCM generally have the ability to capture unstructured data as well.

### Unstructured Components

Unstructured data refers to data elements that do not have a predefined or predetermined form. Unstructured free-text fields in EHRs contain essential clinical detail [17]. These allow medical staff to record the highly variable information that may be medically relevant, and which do not lend themselves easily to structured fields. It is difficult to predict all the fields that may be required ahead of time, or be too demanding for practitioners to fill in numerous individual structured fields.

We offer an example to demonstrate where both structured and unstructured elements are necessary. A discharge summary is a document describing a patient’s course during a single hospital admission. These summaries are often written as detailed narratives, but can also be filled out as templates with parts being auto-populated from other components of the EHR. These summaries can contain features such as diagnoses, allergies, procedures performed, current and prescribed medications, and other relevant information. Unstructured components are found throughout the EHR in other formats as well. This includes nursing notes, which contain nurse assessments and treatments; progress reports, which record relevant events while the patient is under care as well as communication between physicians and other medical staff; consultant reports, which document the specialty consulting details; transfer care reports, anesthesia records, surgery reports, and pathology reports (see Table 2).

**Example Elements of Entered Structured Components:** 

Variable	Description

Discharge Summary	Free-text field describing the patient’s medical history, diagnoses, and events in the hospital deemed relevant by the physician.
Order Summary	Summary of relevant information for every order (test, medication, etc.) including dates and information about the order.
Nursing notes	Nurses’ assessments and descriptions of treatments they provided.
Progress reports	Record of events under care for communication between medical staff, and to chart progress of conditions.
Pathology reports	Diagnoses from pathologists made from examining tissue samples, and descriptions of said tissue.
Admitting Diagnosis	Initial diagnosis a patient was given when admitted.
Allergy notes	Notes about allergic events.

## Understanding the Relationship between Front End and Back End for Research

Previous research on data quality demonstrates that there are potential biases and other issues that need to be accounted for [19-23], and EHR data is no exception. Thus, a researcher must consider the following factors when attempting to design a study using EHR:

1. ***How was data entered?*** The researcher must understand the context of how the data was entered into the system, such as clinical practice variations between units or physician documentation practices; and
2. ***How was care provided?*** The researcher must understand the flow and context of the provided clinical care.

Documentation in EHRs should be thorough and complete, as missing or incorrect information at this stage impacts the quality of downstream data. Data entered by health care workers from the front line are the data that will flow to the back end of the system. Therefore, much of what is entered will be dependent on the clinical context and the clinical practice culture. There can be significant workflow variation between facilities and programs.

Both data entry and coding processes often hinder quality of data obtained downstream. Clinicians entering patient data into an EHR may not document every condition presented, particularly those conditions that are not a primary reason for the visit [19]. For example, depression is often under-coded [20] due to poor documentation if the depression is less severe [21], or if patients feel stigmatized [22]. Similarly, hypertension is often a comorbidity presented by the patient, but the patient may have been admitted due to symptoms of another condition, resulting in undercoding [23]. Following entry of data into the EHRs, clinical coding specialists in health information management departments code patient conditions found in the EHRs using ICD-10-CA. The process of coding health information can also introduce issues of data quality, as some information in the EHR is not required to be coded (secondary conditions that use little to no resources or are not the primary reason for admission), and high demands for productivity sacrifice quality of coding to meet urgent timelines [24].

Within the back end of SCM specifically, the data are stored in raw transactional form, and are left untouched relative to what was entered. The entered data are stored within thousands of tables. Since SCM is a highly normalized database, one cannot always effectively determine if an entire table is trustworthy or not. 

## Data Access and Linkage Considerations

Accessing and linking EHR data presents both technical and privacy-related challenges.

### 1. Technical Considerations

Studies based on relational databases such as EHRs (25) generally require tables to be linked (this includes internal linkage between EHR tables, and external linkage with tables from other databases). Linking these tables requires knowledge of Structured Query Language (SQL). Internal linkage within EHRs is not straightforward, due to the size and complexity. For example, SCM contains over 1,000 tables. Multiple tables and multiple key columns can be attached to a single patient. The hierarchical structure (e.g. visitation) and longitudinal information further complicates linkage process. It is important that the study team incorporate members with expertise in the EHR data structure and in SQL, as well as experts with a thorough understanding of the research question, who can work in close collaboration to extract and link the data.

Another associated challenge is with the process of converting 5.4 million individuals into population cohorts for research studies. This could be achieved by using location-relevant variables within SCM, or by applying data-linkage to other province-wide administrative databases containing resident status information, and then eliminating or sub-setting any non-Calgary residents. The choice to remove non-Calgary residents from the denominator would be dependent on the research question (e.g. if interested in identifying the effect of travel in infectious disease transmissions, such travelers would not be removed).

### 2. Privacy Considerations

A second significant challenge with using EHR data revolves around security, and may require dialogue between health systems, universities, and appropriate stakeholders to move forward. The sensitive nature of EHR data places legal responsibilities on custodians (e.g. AHS in Alberta) for data security. Researchers may have difficulty accessing the data due to required privacy requirements.

Linking patients’ EHR data between multiple internal and external data tables can present an unusual level of privacy risk for both patients and health care providers. EHR free-text data are difficult to anonymize, and may contain identifying information for patients, doctors, nurses, and other health system workers. Moreover, population-level inpatient EHRs such as SCM represent a comprehensive view of the entirety of a patient’s interaction with the health care system. If a large number of tables are linked, it can pose a risk of indirect identification of patients within the data set. Having a specific research question assists in identifying the minimal data elements required from EHR, which in turn can help data custodians de-identify the data to whatever extent is possible. 

## Analytical Approaches, Challenges and Considerations

Analyzing EHR data, and in particular unstructured data, requires non-traditional approaches and technical skills. We will focus on natural language processing and machine learning.

### Analyzing Structured Data

Structured EHR data can be analyzed in multiple ways, including traditional statistical techniques and through machine learning (ML). This section will focus on ML. ML focuses on giving computers the ability to identify patterns in data without being explicitly programmed, inspired by the ability of humans to learn from experience, without being explicitly taught. ML classification algorithms generally can be divided into supervised learning and unsupervised learning. Supervised learning consists of predicting the value of a particular dependent variable (e.g. disease status, length of stay), often called the ‘target’. This is based on the given values of a number of independent variables or ‘features’ (e.g. age, sex, diagnosis codes), together with a number of training examples in which the correct value of the target is manually assigned by a person. These manually assigned values are called ‘training labels’. Unsupervised learning refers to situations in which no training labels are available (not commonly done in analysis of EHR data). Machine learning, in this case, extends into deep learning, which is a state-of-the-art method that has led to its exploration usage in EHRs [16]. Deep learning methods do not require expert knowledge or pre-defined rules, as the hidden manifold can be learned from big data. 

### Analyzing Unstructured Data

Natural language processing (NLP) allows machines to identify the structure (syntax) and extract the meaning (semantics) of human language. NLP is primarily useful in the EHR context when processing free-text unstructured data elements. An important part of NLP is part-of-speech tagging (determine whether one word is a noun, verb, adjective, etc.), negation detection, and sentence boundary detection. This facilitates searches for clinical concepts in unstructured EHR components.

The Unified Medical Language System (UMLS) is one example of an NLP system [26]. The clinical Text Analysis and Knowledge Extraction System (cTAKES) is another example of an open-source Natural Language Processing system [27]. cTAKES included pre-trained machine learning algorithms specifically designed for clinical texts. The hybrid system, which combined cTAKES and expert knowledge decision rules, became state-of-the-art, up until deep learning was invented. Deep learning and word embedding have become two cornerstones of modern NLP.

### Challenges of Analyzing EHR Data in SCM

Traditional methods are unable to handle large numbers of features and unstructured data; however, machine learning can handle both. There are three major analytical challenges associated with these techniques. First, trained experts in ML and data science are needed. Second, a large number of records is required, and computational requirements must be met. Third, is it challenging to interpret the models, and requires specific expertise. Finally, quality of data entered from the front end (as discussed previously) can cause issues in the data downstream.

As previously discussed, EHR data is very heterogeneous, and must be accounted for when determining appropriate techniques. Therefore, one must have sufficient understanding of the data, as well as possess the technical skills to conduct ML and NLP. There are many open, online courses available for technical training, and many universities are now establishing graduate training programs.

 ML requires large amounts of data and often is challenging to interpret. Deep learning, a subfield within ML, can offer better performance than machine learning, but requires even more data and can be more challenging to interpret. The sample size of the study must be large enough to partition the data into training, validation, and test sets. Generally, the training set should be given the largest portion of the sample, which is a decision that is also influenced by the size of the total dataset. ML algorithms require gold-standard labels for algorithms to train on if supervised learning is used. Chart review is the usual gold standard to validate data in health research, but can be expensive and time-consuming.

In addition to having sufficiently large data and the required skill-sets, hardware computational requirements (e.g. Graphics Processing Unit cluster for deep learning) must be met to conduct such analyses. Researchers should note that EHR-related privacy requirements might hamper data transfer to hardware.

A major criticism of ML and deep learning is that the models can be difficult to interpret. Achieving interpretability is currently an active area of research within computer sciences, and there are some ML techniques that are easier to interpret than others. Furthermore, the context of the problem also determines whether certain processes need to be interpretable or not. For example, if a researcher is interested in whether someone has a disease (i.e. case definition) using a huge data volume, then achieving high predictive accuracy may be more important than precisely understanding the causal chain. EHRs contain huge volumes of data for each patient, sometimes beyond what traditional techniques can process. ML and deep learning are therefore sound methodologies for EHR research, as long as research objectives align with the purposes of the techniques.

## Example Applications of the EHR: Developing learning algorithms for risk adjustment analysis to achieve precision medicine and precision public health

The potential for EHR for clinical research applications have been described previously [28]. Researchers have used EHR data to provide real-time adverse surgical event reporting [29], recruit participants for clinical trials [30], build systems to automatically infer medical problems [31], and for pharmacoepidemiology and public health surveillance [32, 33]. A population-wide inpatient EHR (such as SCM) can be used to facilitate local and regional healthcare system planning in addition to clinical research. Alberta’s health care is structured as a single payer system, which is under AHS. This structure allows the creation of a system-wide data repository for provincial planning. The crux of health system planning requires accurate and timely risk adjustment analysis. Risk adjustment aims to identify patient health risks, and build models that compare, adjust and predict/forecast associated health expenditure or outcomes of interest [34]. The principles of risk adjustment analysis using EHR data is therefore a critical component of precision medicine, as it would lead to better patient outcomes and improved health system planning and management.

It should be noted that inpatient EHR systems, such as SCM, provide granular clinical details and may lack such detail on non-clinical information, such as school achievements, patient complaints, and so forth. Therefore, data linkage between multiple population-level data sources is required to achieve precision medicine and precision public health. Identifying appropriate data sources for population data linkage is then dependent on the context of the research question. We aim to explore data linkage with non-inpatient clinical settings, such as primary care data and non-clinical population databases, within Alberta. 

## Conclusion and Next Steps

EHR data are potentially an optimal data source for research. Clinical details, which are not readily available in administrative data, can be augmented with the data extracted from EHR. Utilizing EHR will lead to improved case definitions and identification of conditions, leading to development of robust risk adjustment methodologies. This will allow the creation of personalized outcome predictions/comparisons, which constitutes the core principles of precision medicine. There are administrative and analytical challenges associated with EHR data. However, these challenges are surmountable and worth overcoming. EHR data have led to the use of sophisticated analytical techniques such as machine learning and natural language processing.

The Center for Health Informatics (CHI) at the University of Calgary was established to work with EHR and other data types in pursuit of health data science. The CHI brings together Albertan stakeholders (e.g. UofC, AHS, Ministry of Health (Alberta Health), and Alberta Strategy for Patient Oriented Research (SPOR)) to allow the EHR access for research use under a controlled environment. Our team at the CHI has completed chart review for 3,000 randomly selected inpatients admitted in Calgary hospitals. We are utilizing SCM EHR and other data (e.g. administrative data, clinical registry and chart review data) to develop and validate case definition algorithms, ultimately improving research methods such as risk adjustment. Ultimately, harnessing the full potential of EHR data can lead to better patient outcomes and system improvements.

## Acknowledgments

The authors thank Kevin Lonergan, Sang Ming Lee, Jason Jiang, and Dr. Abdel Aziz Shaheen for assistance with preparation of this manuscript.

## Ethics Statement

This article is based on data from human subjects and no animal subjects. All authors have read the manuscript, agree the work is ready for submission to the journal, accept responsibility for the manuscript’s contents, and have no biomedical, financial or other potential conflicts of interest. The work of this article has received ethics approval from University of Calgary's Conjoint Health Research Ethics Board (REB19-0088).

## Abbreviations

EHR	Electronic Health Records
SCM	Sunrise Clinical Manager
AHS	Alberta Health Services
HL7	Health Level 7
UMLS	Unified Medical Language System
SNOMED	Systematized Nomenclature of Medicine
NLP	Natural Language Processing
MELD	Model for End-Stage Liver Disease
SDES	Structured Data Entry Systems
EF	Ejection Fraction
CHI	The Center for Health Informatics
SPOR	Strategy for Patient Oriented Research
UofC	University of Calgary
SQL	Structured Query Language
PHN	Provincial Healthcare Number
